# Complications of Endoscopic Pressure Study Integrated System: Review of 1205 Cases in 10 Years’ Experience

**DOI:** 10.1002/deo2.70173

**Published:** 2025-07-16

**Authors:** Miyuki Iwasaki, Ippei Tanaka, Haruhiro Inoue, Masachika Saino, Kei Ushikubo, Kazuki Yamamoto, Yohei Nishikawa, Kaori Owada, Satoshi Abiko, Mayo Tanabe

**Affiliations:** ^1^ Digestive Diseases Center Showa University Koto Toyosu Hospital Tokyo Japan

**Keywords:** complication, endoscopic pressure study integrated system, intragastric pressure, micro‐mucosal bleedings, superficial mucosal tears

## Abstract

**Background and Aims:**

The Endoscopic Pressure Study Integrated System (EPSIS) is a novel endoscopic tool to assess lower esophageal sphincter (LES) function by monitoring intragastric pressure (IGP) through insufflation. While previous studies have confirmed its diagnostic utility for LES dysfunction‐related disorders such as gastroesophageal reflux disease and achalasia, there have been no reports evaluating its safety. Thus, this study aimed to assess its complication rates and detailed characteristics.

**Methods:**

We retrospectively reviewed 1205 consecutive EPSIS cases performed between December 2015 and September 2024 at our institution. Cases with adverse events (AEs) were identified, and clinical characteristics, waveform patterns, IGP parameters, and need for additional treatment were analyzed.

**Results:**

AEs occurred in 35 cases (2.9%), including 32 cases (2.7%) of micro‐mucosal bleedings (MMBs) and three cases (0.25%) of superficial mucosal tears (SMTs). All cases were minor (Clavien‐Dindo grade I), without the need for any additional intervention. All 35 cases exhibited an uphill waveform. SMTs were associated with pressure spikes due to vomiting or belching. No serious AEs, including perforations or active bleeding, were observed.

**Conclusions:**

EPSIS is a safe and minimally invasive procedure, with low complication rates comparable to routine upper endoscopy. To maintain its safety, EPSIS should be performed under sedation with CO₂ insufflation. In addition, insufflation during EPSIS should be controlled so that the IGP is maintained less than 25 mmHg. However, this threshold may vary depending on the patient's underlying conditions or clinical background; therefore, further research is warranted.

**Clinical Trial Registration:**

N/A.

## 1 Introduction

The Endoscopic Pressure Study Integrated System (EPSIS) is a novel endoscopic tool for assessing lower esophageal sphincter (LES) function by monitoring intragastric pressure (IGP) during gastric insufflation [1]. Previous studies have reported that EPSIS is useful in the diagnosis of LES dysfunction‐related disorders such as gastroesophageal reflux disease (GERD) and achalasia [2, 3, 4]. As with conventional endoscopic examinations, careful attention to over‐insufflation and patient conditions is essential to ensure procedural safety. Although EPSIS is generally considered safe, we have encountered a number of cases in which minor bleeding or shallow mucosal tears occurred during the procedure. However, the exact incidence, characteristics, and clinical significance of these findings have not been thoroughly investigated. Therefore, this study aims to evaluate the safety of EPSIS and identify key considerations for preventing complications.

## 2 Methods

### 2.1 Study Design and Patients

We reviewed cases of EPSIS performed at our institution using digital medical records. Cases in which adverse events (AEs) occurred during EPSIS were extracted, and we retrospectively analyzed patient parameters, EPSIS waveforms, maximum IGP (IGP max), and the necessity for additional treatment or follow‐up examinations. In this study, minor bleeding and shallow tears observed during EPSIS were defined as micro‐mucosal bleedings (MMBs) and superficial mucosal tears (SMTs), respectively. MMBs were defined as punctate or patchy mucosal bleeding without visible mucosal disruption. SMTs, which are generally categorized as Mallory–Weiss tears (MWTs), were extremely shallow in our cases and did not meet the typical clinical severity of MWTs. Therefore, we used the separate term SMTs, defined as mucosal tears confined to the lamina propria without active bleeding. For cases with MMBs, we assessed the bleeding site, while for cases with SMTs, the lesion location was classified using the Zeifer classification for MWTs [5]. The follow‐up period after EPSIS was conducted in accordance with the standard protocol for post‐endoscopic procedures, unless specific complications or concerns necessitated additional monitoring.

The primary outcome was the incidence of EPSIS‐related complications. The secondary outcome was the identification of procedural factors associated with their occurrence during the procedure.

This study was conducted in accordance with the Declaration of Helsinki and was approved by the Institutional Review Board of Showa University Hospital (approval number: C‐T2025‐0573). Informed consent was obtained from all patients prior to undergoing EPSIS. Regarding conflict of interests, author IH is an advisor for Olympus Corporation and Top Corporation. He has also received educational grants from Olympus Corporation and Takeda Pharmaceutical Co. The other authors have no conflicts of interest to disclose. All procedures were performed consecutively following routine esophagogastroduodenoscopy (EGD).

### 2.2 EPSIS Procedure

**FIGURE 1:**
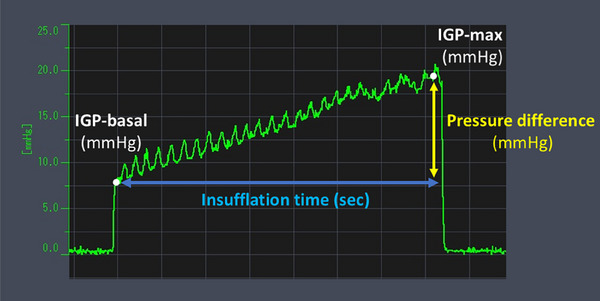
Waveform pattern and intragastric pressure parameters of the endoscopic pressure study integrated system.

EPSIS measurements were performed using computer with pressure measurement device (Figure S1), high‐definition endoscopes (GIF‐H290Z and GIF‐XZ1200; Olympus Corp., Tokyo, Japan), a CO₂ insufflator (Olympus Corp.), and a high‐flow tube (MAJ‐1741; Olympus Corp.), maintaining a constant CO₂ flow of approximately 1.5 L/min. Intravenous propofol was administered for sedation in all cases. The tip of a disposable irrigation tube (AF‐WT; Forte Grow Medical Corp., Tochigi, Japan) was connected directly to the working channel cap (Figure S2), and its other end was linked to a pressure measurement device (TR‐W550, TR‐TH08, and AP‐C35; Keyence, Osaka, Japan). Insufflation was performed under direct endoscopic visualization in the retroflexed view (Figure S3). We recorded the waveform pattern and IGP parameters, including basal IGP, IGP max, and insufflation time (Figure 1). IGP max was defined as the maximum pressure of consecutive IGP measurements. Since IGP could be monitored in real time during gastric insufflation, the procedure was performed with careful attention to ensure that the IGP max did not exceed 25 mmHg, which was set as a procedural safety margin. All EPSIS procedures were performed by endoscopists with experience in more than 1000 upper gastrointestinal endoscopic examinations.

### 2.3 Statistical Analysis

Associations between MMBs, patient characteristics, and underlying diseases were evaluated statistically. Categorical data were expressed as the number and percentage, whereas continuous data were expressed as the median and interquartile range (IQR). Group comparisons were performed using the t‐test for continuous variables and Fisher's exact test or Pearson's chi‐square test for categorical variables. All analyses were conducted using EZR version 1.65 (Saitama Medical Center, Jichi Medical University), a graphical user interface for R designed for biostatistical applications (http://www.jichi.ac.jp/saitama‐sct/SaitamaHP.files/statmed.html). A *p*‐value of <0.05 is considered statistically significant. All authors had full access to the data and approved the final version of the manuscript.

## 3 Results

Between December 2015 and September 2024, a total of 1205 patients underwent EPSIS at our institution. Table 1 summarizes the clinical characteristics of patients. The aim of EPSIS was as follows: evaluation of reflux symptoms (*n* = 445, 36.9%), evaluation of dysphagia (*n* = 327, 27.1%), and post‐treatment evaluation (*n* = 342, 28.4%). The category of post‐treatment evaluation was further subdivided into three groups: post‐laparoscopic anti‐reflux surgery (*n* = 69, 5.7%), post‐endoscopic anti‐reflux intervention (*n* = 161, 13.4%), and post‐peroral endoscopic myotomy (POEM) (*n* = 131, 10.9%). Laparoscopic anti‐reflux surgery includes Nissen and Toupet fundoplication. In contrast, endoscopic anti‐reflux intervention includes anti‐reflux mucosectomy (ARMS) and anti‐reflux mucosal ablation. The prevalence of major esophageal and gastric findings was as follows: GERD (*n* = 443, 36.8%), achalasia (*n* = 299, 24.8%), hiatal hernia (*n* = 261, 21.7%), eosinophilic esophagitis (*n* = 26, 2.2%), and atrophic gastritis (*n* = 157, 13.0%). AEs occurred in 35 cases (2.9%), including 32 cases (2.7%) of MMBs and three cases (0.25%) of SMTs, as shown in Table 2. These events were identified during or immediately after EPSIS. No delayed AEs were reported. All patients were instructed to contact the hospital in case of post‐procedural symptoms such as abdominal pain, but no such cases were recorded.

**TABLE 1:** Patients' clinical characteristics

Patients' characteristics (*n* = 1205)	
Age, years, median (range)	54 (13–95)
Sex, male:female (male %)	705:500 (58.5)
Antiplatelet or anticoagulation use, *n* (%)	27 (2.2)
The aim of EPSIS, *n* (%)	
Evaluation of reflux symptoms	445 (36.9)
Evaluation of dysphagia	327 (27.1)
Post‐treatment evaluation	
Post‐laparoscopic anti‐reflux surgery	69 (5.7)
Post‐endoscopic anti‐reflux intervention	161 (13.4)
Post‐POEM	131 (10.9)
Others	136 (11.3)
Endoscopic finding, *n* (%)	
Gastroesophageal reflux disease	443 (36.8)
Esophageal achalasia	299 (24.8)
Hiatal hernia	261 (21.7)
Eosinophilic esophagitis	26 (2.2)
Atrophic gastritis	157 (13.0)
Open type	52 (4.3)

Abbreviations: EPSIS, endoscopic pressure study integrated system; POEM, per‐oral endoscopic myotomy.

**TABLE 2:** Potential adverse events during the endoscopic pressure study, integrated system, and observed incidence.

Potential adverse events	Number of events, incidence rate (%)
Bleeding	
Micro‐mucosal bleedings (MMBs)	32 (2.7)
Major bleedings	0 (0)
Superficial mucosal tears (SMTs)	3 (0.25)
Mallory‐Weiss tears	0 (0)
Perforation	0 (0)
Infection	0 (0)
Air embolism	0 (0)
Subcutaneous emphysema	0 (0)
Pneumothorax	0 (0)
Abdominal compartment syndrome	0 (0)

### 3.1 Micro‐mucosal Bleedings

**FIGURE 2:**
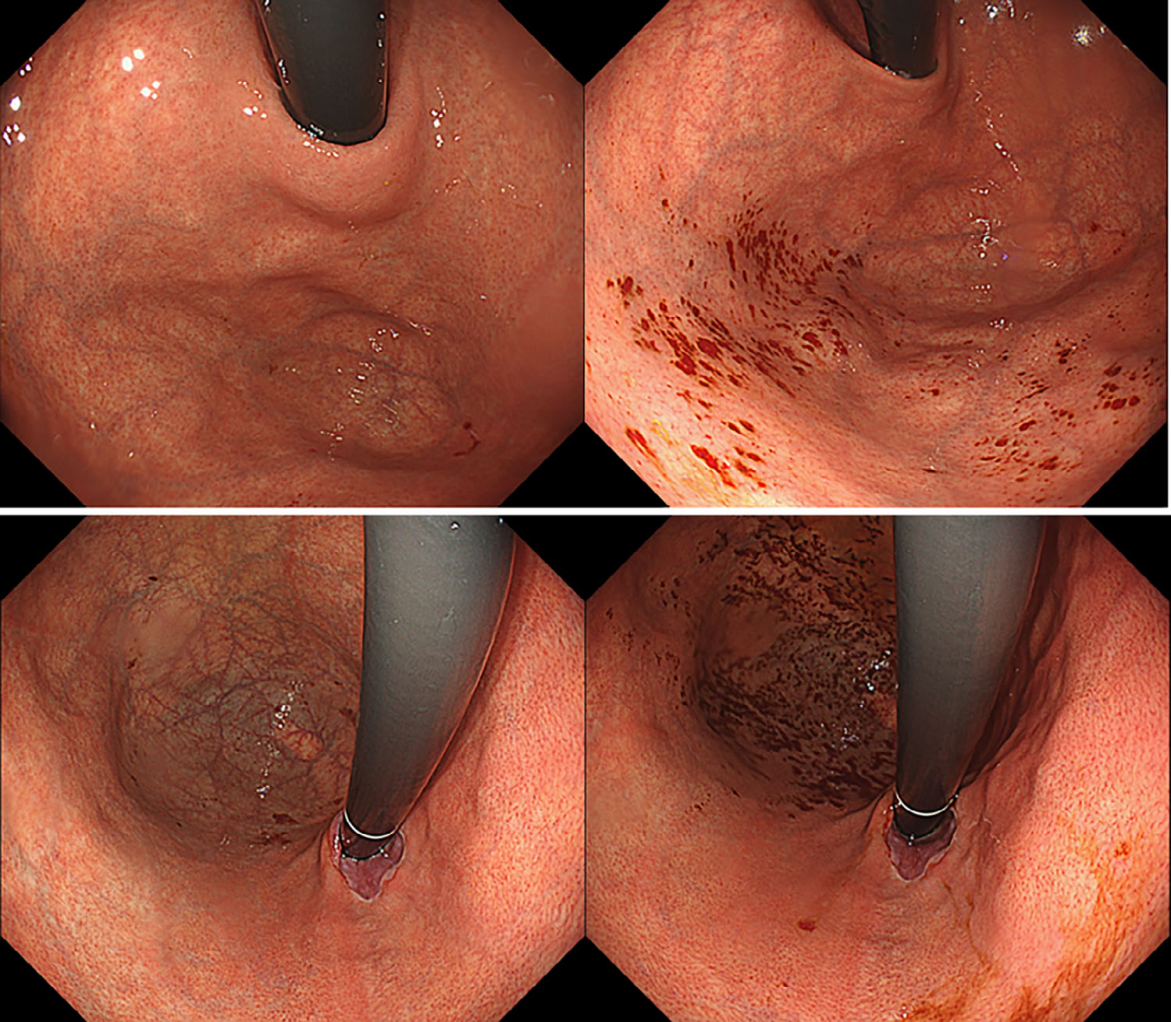
Cases of micro‐mucosal bleedings in the gastric fundus.

**FIGURE 3:**
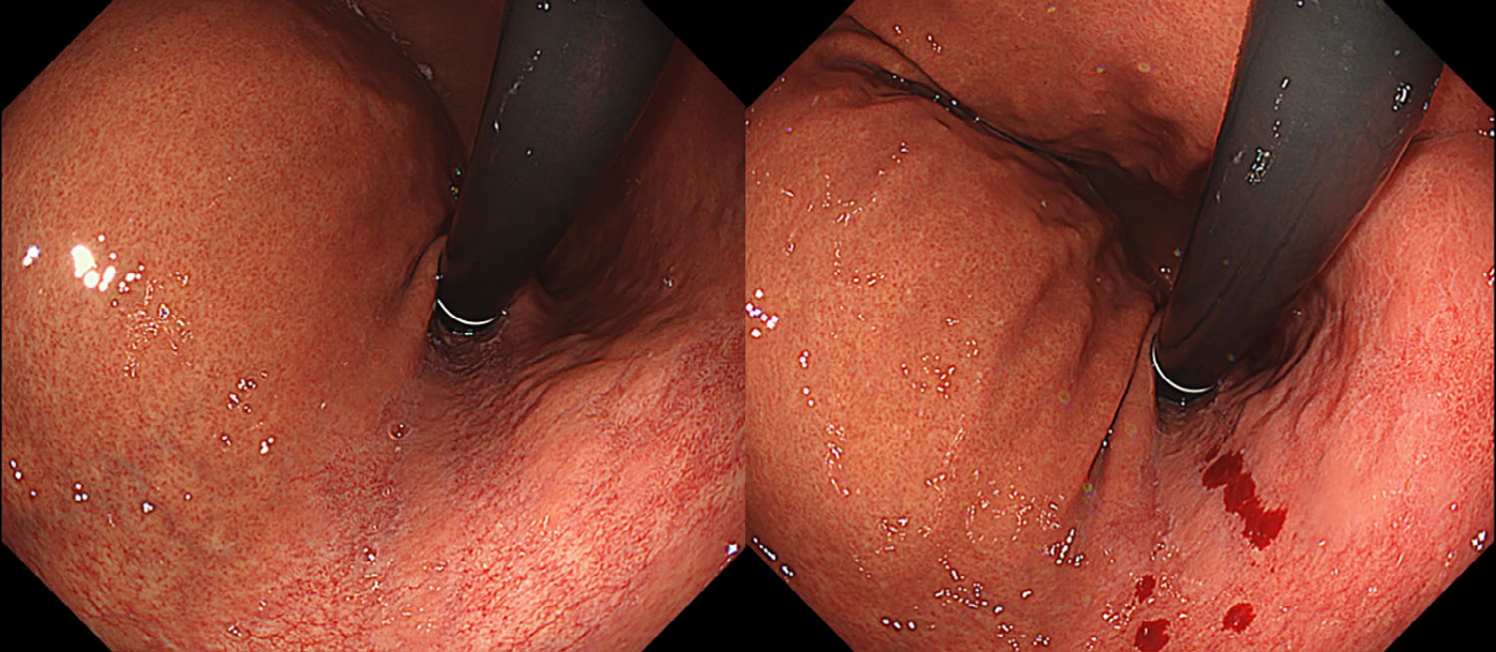
A case of micro‐mucosal bleedings arising in the atrophic mucosa of the lesser curvature of the gastric body.

**FIGURE 4:**
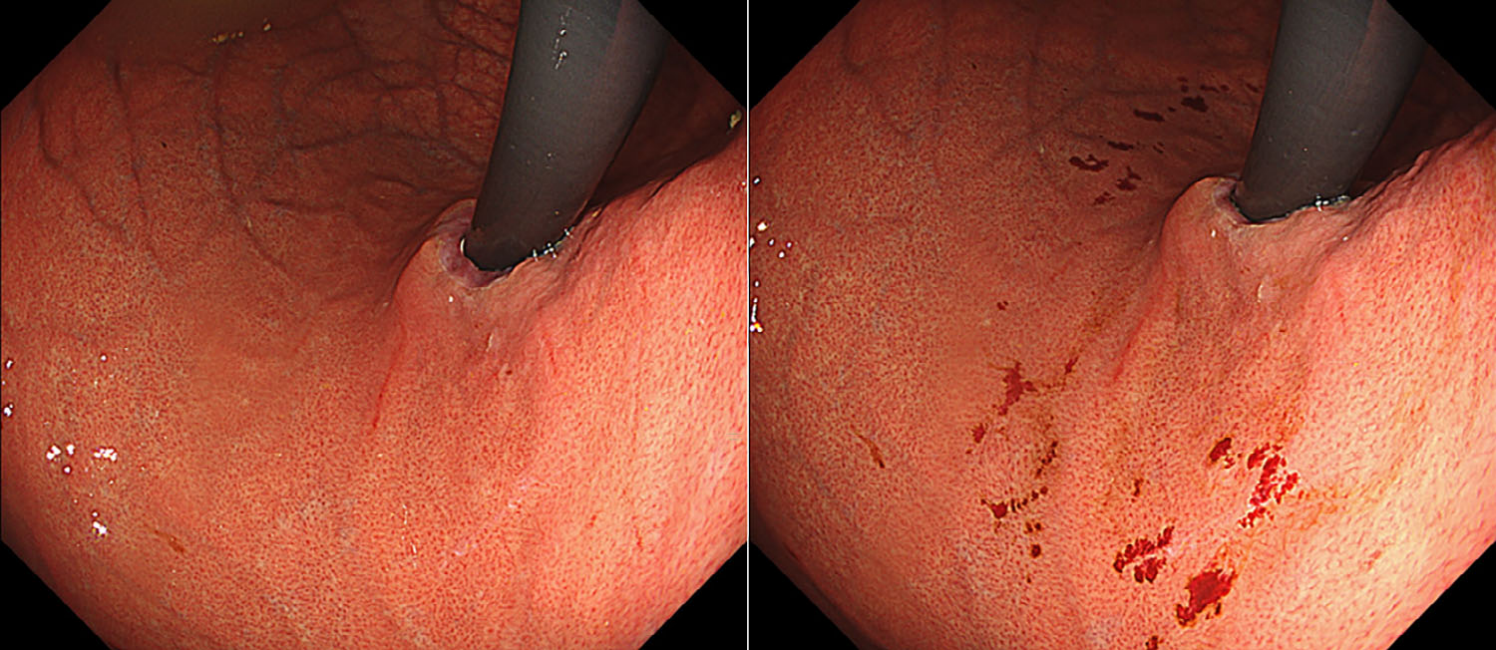
A case of micro‐mucosal bleedings arising from the gastric fundus to the lesser curvature of the upper gastric body in a patient with esophageal achalasia.

Table 3 presents the clinical characteristics of patients with micro‐mucosal bleedings (MMBs). In all cases, spontaneous hemostasis was observed, and no additional treatment or examination was required. The median age was comparable to the overall cohort (54 vs. 53 years), with a slightly higher proportion of female patients. One patient (3.1%) was on anticoagulant therapy. The aim of EPSIS was as follows: evaluation of reflux symptoms (*n* = 17, 53.1%), evaluation of dysphagia (*n* = 10, 31.3%), post‐laparoscopic anti‐reflux surgery (*n* = 1, 3.1%), post‐endoscopic anti‐reflux intervention (*n* = 0, 0%), post‐POEM (*n* = 2, 6.3%). Statistical analysis revealed that female sex was significantly associated with MMBs (59.4% in the MMB group vs. 41.0% in the non‐MMB group, *p* = 0.0457). In contrast, endoscopic anti‐reflux intervention history (0% vs. 13.4%, *p* = 0.0159) and GERD diagnosis (3.1% vs. 37.7%, *p* < 0.001) were significantly less common in the MMB group. Regarding gastric atrophy, no significant difference was found between patients with and without atrophy (21.9% vs. 12.8%, *p* = 0.359). However, patients classified as open‐type according to the Kimura–Takemoto classification had a significantly higher incidence of MMBs compared to those without atrophy (15.6% vs. 12.8%, *p* = 0.0321). All 32 MMB cases showed an uphill waveform pattern, with a mean IGP max of 21.7 mmHg. Bleeding sites were identified as the gastric fundus in 17/19 cases (89.5%) of the patients who have esophageal lesions (GERD, achalasia, hiatal hernia, or eosinophilic esophagitis) and the gastric lesser curvature in 7/7 cases (100%) of the patients who have atrophic gastritis (Figures 2, 3, 4).

**TABLE 3:** Patient clinical characteristics of micro‐mucosal bleeding (MMBs).

	MMBs (*n* = 32)	Non‐MMBs (*n* = 1173)	*p*‐Value
Age, years, median (range)	53 (23–78)	54 (13–95)	0.277
Sex, male: female (male %)	13:19 (40.6)	692:481 (59.0)	0.0457^*^
Antiplatelet or anticoagulation use, *n* (%)	1 (3.1)	26 (2.2)	0.578
The aim of EPSIS, *n* (%)			
Evaluation of reflux symptoms	17 (53.1)	428 (36.5)	0.149
Evaluation of dysphagia	10 (31.3)	317 (27.0)	0.845
Post‐treatment evaluation			
Post‐laparoscopic anti‐reflux surgery	1 (3.1)	68 (5.8)	0.716
Post‐endoscopic anti‐reflux intervention	0 (0)	161 (13.7)	0.0159^*^
Post‐POEM	2 (6.3)	129 (11.0)	0.568
Others	2 (6.3)	134 (11.4)	‐
Endoscopic finding, n (%)			
Gastroesophageal reflux disease	1 (3.1)	442 (37.7)	<0.001^*^
Esophageal achalasia	9 (28.1)	290 (24.7)	1.000
Hiatal hernia	9 (28.1)	252 (21.5)	0.675
Eosinophilic esophagitis	1 (3.1)	25 (21.3)	0.548
Atrophic gastritis	7 (21.9)	150 (12.8)	0.359
Open type	5 (15.6)	47 (4.0)	0.0321^*^
EPSIS parameters			
Uphill waveform, *n* (%)	32 (100)	‐	‐
Mean IGP max, mmHg (SD)	21.7 (2.39)	‐	‐

* *p* < 0.05, comparison of the MMBs and the non‐MMBs groups.

Abbreviations: BMI, body mass index; EPSIS, endoscopic pressure study integrated system; IGP, intragastric pressure; MMBs, micro‐mucosal bleedings; POEM, per‐oral endoscopic myotomy; SD, standard deviation.

### 3.2 Superficial Mucosal Tears

**FIGURE 5:**
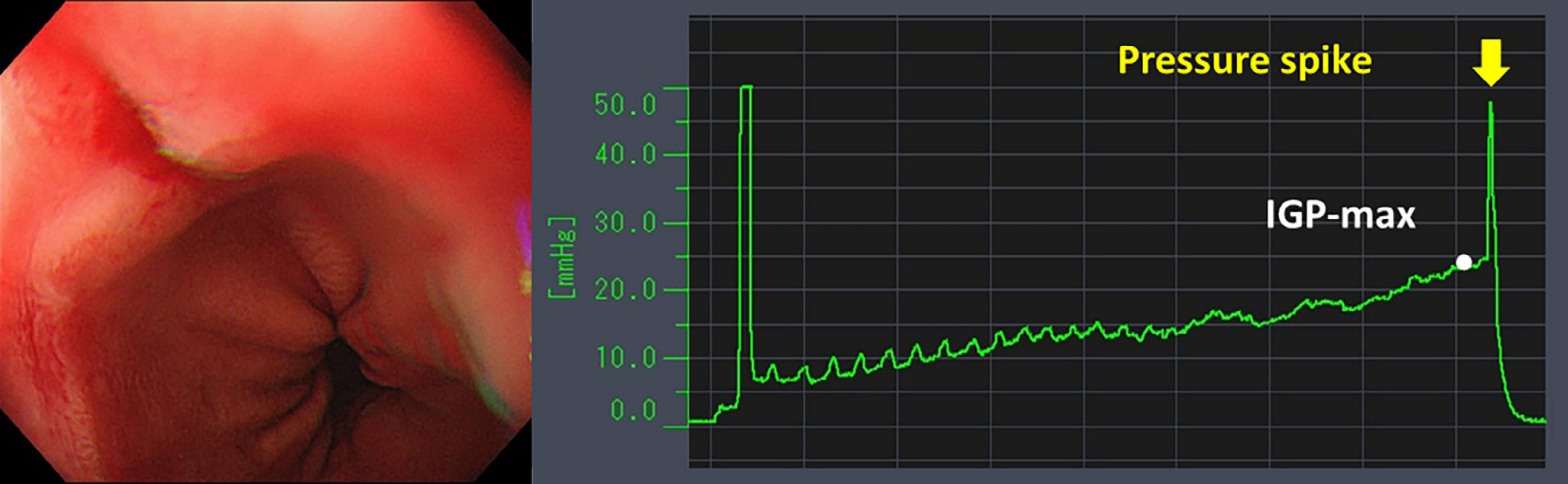
A case of Zeifer classification type III superficial mucosal tear (SMT) (Case 1 in Table 4). The endoscopic pressure study integrated system waveforms were an uphill pattern, and the maximum intragastric pressure was 23.5 mmHg. A momentary pressure spike was observed during air insufflation. SMT was observed in the 11 o'clock direction of the gastroesophageal junction.

**FIGURE 6:**
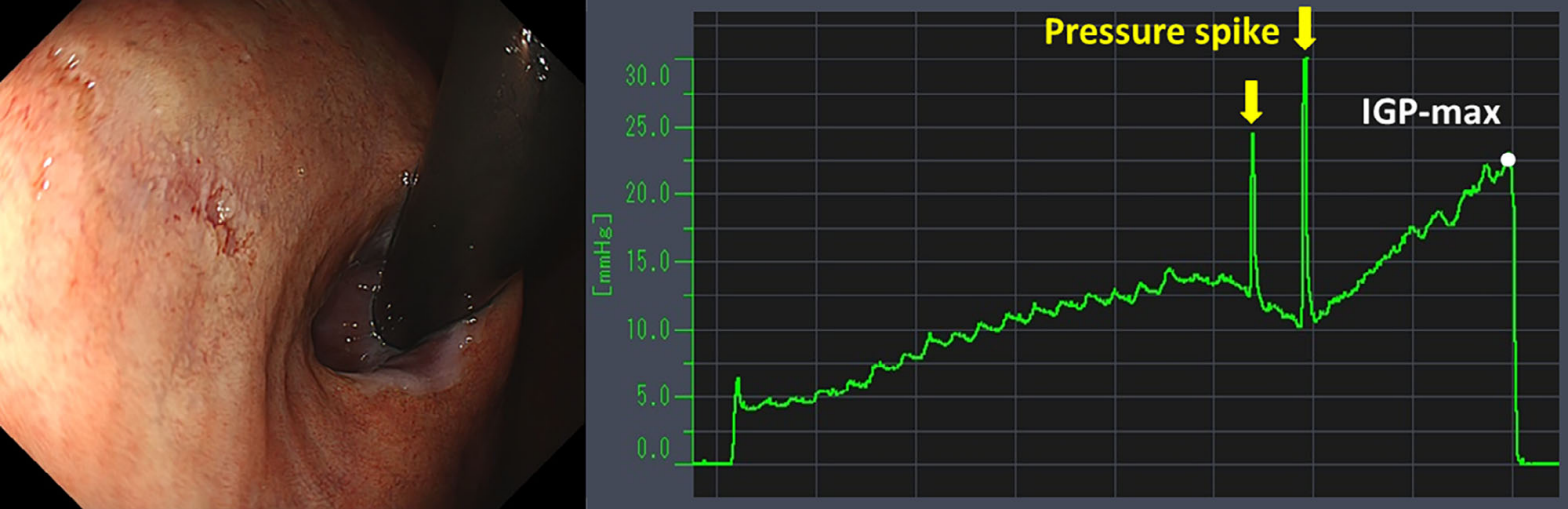
A case of Zeifer classification type II superficial mucosal tear (SMT) (Case 2 in Table 4). The endoscopic pressure study integrated system waveforms were an uphill pattern, and the maximum intragastric pressure was 23.1 mmHg. Two pressure spikes were observed during air insufflation. SMT was observed in the upper lesser curvature of the gastric body.

**FIGURE 7:**
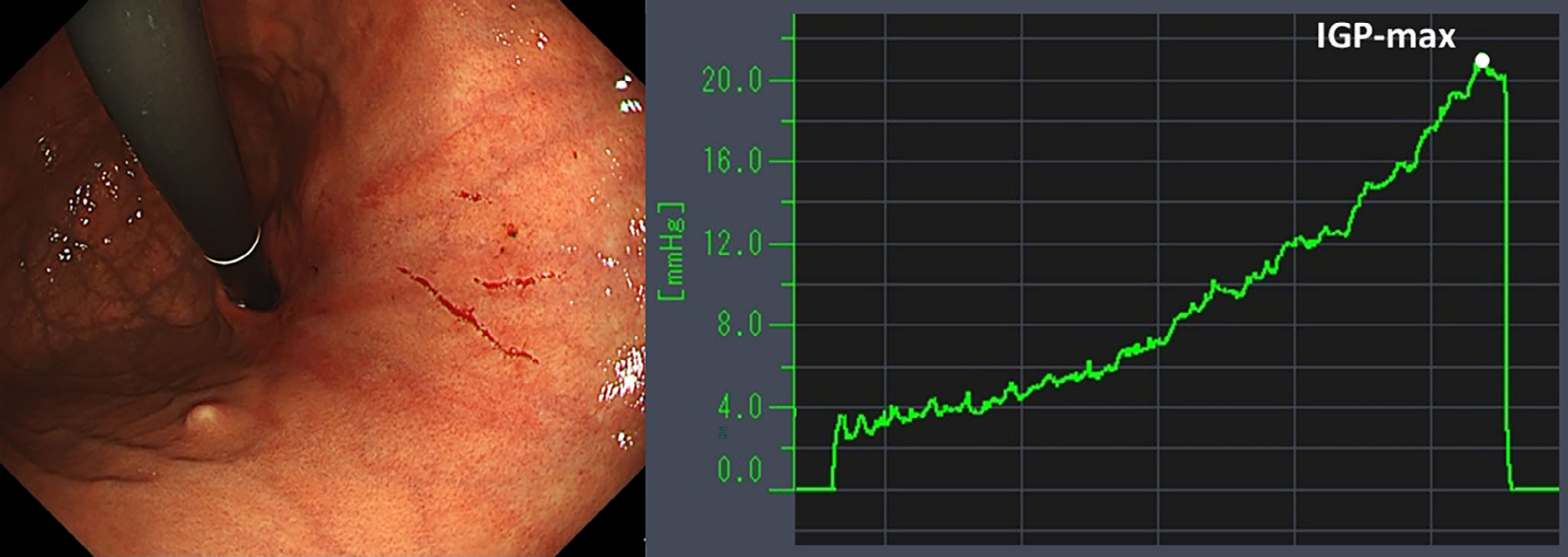
A case of Zeifer classification type II superficial mucosal tear (SMT) of the patient after anti‐reflux mucosectomy (Case 3 in Table 4). The endoscopic pressure study integrated system waveforms were an uphill pattern, and maximum intragastric pressure was 20.9 mmHg. SMT was observed in the upper lesser curvature of the gastric body.

Table 4 details the cases of SMTs. The purpose of EPSIS was to examine two patients for reflux symptoms and one patient during follow‐up after ARMS for reflux esophagitis. The EPSIS waveforms of the two patients with symptoms were an uphill pattern, and IGP max was 23.5 mmHg and 23.1 mmHg, respectively (Figure 5 corresponds to Case 1 in Table 4, and Figure 6 corresponds to Case 2). In both cases, a momentary pressure spike induced by the vomiting reflex or rapid belching was observed during air insufflation. After confirming the pressure spike, the operator stopped the insufflation immediately. However, Zeifer classification type III (esophagogastric coexisting type) SMTs were observed in the 11 o'clock direction of the gastroesophageal junction (GEJ), and in the other case, Zeifer classification type II (gastric localized type) SMTs were observed in the upper lesser curvature of the gastric body. The EPSIS waveform of the patient after ARMS was an uphill pattern, and IGP max was 20.9 mmHg (Figure 7 corresponds to Case 3 in Table 4). During insufflation, Zeifer classification type II SMTs were observed in the middle lesser curvature of the gastric body under direct endoscopic view, and the insufflation was promptly stopped. No pressure spike of the IGP was observed. All three EPSIS procedures were performed under moderate sedation with propofol by endoscopists with experience in over 1000 EGDs. All cases demonstrated an uphill pattern waveform, with IGP max values of 20.9–23.5 mmHg. The duration of each procedure is summarized in Table 4. Pressure spikes triggered by vomiting reflexes or rapid belching were observed in two cases (case 1 and case 2), leading to SMTs. In cases 1 and 2, vomiting reflexes occurred during endoscope insertion, and additional doses of anesthetic were administered as required throughout the procedure. In all cases, the tear was confined to the mucosal layer with no exposure of the muscle layer confirmed by endoscopic observation. Bleeding was minimal, and spontaneous hemostasis was achieved during observation, and no additional treatment or further testing was required.

**TABLE 4:** Patient clinical characteristics of superficial mucosal tears (SMTs).

								EPSIS parameters					
No.	Age	sex	BMI (kg/m^2^)	Anticoagulant	The aim of EPSIS	History of fundoplication treatment	Endoscopic findings	waveform	IGP max (mmHg)	Spike	Insufflation time (s)	MMBs	SMTs
1	29	M	22.4	‐	Evaluation of reflux symptoms	‐	Hiatus hernia	Uphill	23.5	+	76	‐	+
2	69	M	22.3	‐	Evaluation of reflux symptoms	‐	Atrophic gastritis	Uphill	23.1	+	74	+	+
3	42	F	15	‐	Post‐treatment evaluation	ARMS	Gastric scar after ARMS treatment	Uphill	20.9	‐	28	‐	+

Abbreviations: ARMS, anti‐reflux mucosectomy; BMI, body mass index; EPSIS, endoscopic pressure study integrated system; IGP, intragastric pressure; MMBs, micro‐mucosal bleedings; SMTs, superficial mucosal tears.

## 4 Discussion

In this retrospective review of 1,205 EPSIS cases, we identified 32 cases (2.7%) of MMBs and three cases (0.25%) of SMTs, all of which were minor, without the need for any intervention. Although EPSIS is a type of stress test that evaluates LES function by increasing IGP with air insufflation, the complication rate of SMTs (0.25%) was comparable to that of routine EGD, and no other complications were observed.

Complications of EGD include bleeding, perforation, and infection, such as aspiration pneumonia [6]. Extremely rare complications related to insufflation include air embolism, subcutaneous emphysema, pneumothorax, and abdominal compartment syndrome [7]. The causes of these complications are suggested to be related not only to insufflation during endoscopy but also to several factors, such as biopsy‐induced mucosal injury or perforation.

In our EPSIS cases, only MMBs and SMTs were observed, and all were classified as Clavien‐Dindo grade I [8]. MMBs are an occasionally observed finding related to insufflation and the vomiting reflex during endoscopic observation. These findings most commonly appear simultaneously and diffusely in the fornix and along the lesser curvature of the gastric body. In our study, bleeding was more frequently observed in the gastric fundus among patients with esophageal disorders, and in the lesser curvature of the gastric body among those with atrophic gastritis; however, the underlying mechanisms for these site‐specific distributions remain unclear. In this study, MMBs were significantly more frequent in female patients and those with open‐type atrophic gastritis, whereas they were significantly less frequent in patients with a history of endoscopic anti‐reflux intervention and those with GERD. It is well recognized that patients with atrophic gastritis tend to have thinning of the gastric mucosa [9]. Furthermore, previous studies have reported that females tend to have thinner gastric mucosa than males [10], suggesting that mucosal thickness may contribute to the susceptibility to MMBs. In patients with GERD, the development of MMBs may be less likely due to a reduced increase in IGP. As for the lower incidence of MMBs in patients with a history of endoscopic anti‐reflux intervention, it is possible that endoscopists might have limited insufflation during the examination due to the postoperative status. However, further investigation is warranted to clarify these mechanisms. Since they are not clinically significant bleeds (ASGE defined as a hemoglobin drop >2 g/dL and/or evidence of hematemesis, melena, or hematochezia [11]), they are not usually considered complications in EGD, and the frequency and risk factors have not been studied to date. Also, in our study, MMBs were not accompanied by active bleeding and did not require any additional intervention. Therefore, it may be controversial whether MMBs should be regarded as complications of EPSIS, and they could instead be defined as minor mucosal “changes”. However, MMBs are the initial findings that can often be observed during routine screening endoscopy when excessive insufflation is applied. Considering that further insufflation may lead to clinically significant complications such as mucosal tears, we believe it may be reasonable to include MMBs among EPSIS‐related complications.

On the other hand, SMTs may be considered a mild variant of MWTs. MWTs are known as mucosal tears of the GEJ, first reported by George Mallory and Soma Weiss in 1929 [12]. MWTs are known as a cause of upper gastrointestinal bleeding, often due to repetitive vomiting in heavy alcohol drinkers, but also a complication of EGD, with an incidence of 0.35% [13, 14]. Risk factors for MWTs include older age, BMI <18.5, and atrophic gastritis [15, 16, 17]. In our study, the SMT cases were characterized by the presence of a pressure spike and a history of previous treatment with ARMS, but further accumulation and analysis of cases are needed to identify potential risk factors.

EPSIS waveforms correlate with anti‐reflux barrier (ARB) function. Patients with preserved ARB function tend to have higher IGP max and an uphill pattern, while patients with impaired ARB function tend to have low IGP max and a flat pattern. It was reported that flat pattern waveform and IGP max <18.7 mmHg were associated with GERD [2]. Notably, all MMB/SMT cases had an uphill pattern, with 33/35 cases exceeding 18.7 mmHg. Therefore, GERD patients may have a lower risk of mucosal injury during EPSIS, as suggested by the findings in cases with MMTs. With regard to the upper limit of IGP during the EPSIS procedure, the findings of this study suggest that IGP should ideally be maintained below 25 mmHg. This recommendation is based on the fact that no severe AEs and only 2 % minor complications occurred when IGP max was kept under 25 mmHg. Specifically, the mean IGP max in cases with MMBs was 21.7 mmHg, while in cases with SMTs, the IGP max ranged from 20.9 to 23.5 mmHg. These findings indicate that deflation should be started once the IGP exceeds 20 mmHg, and that 25 mmHg should be regarded as a safety threshold.

To prevent a pressure spike, which was a major cause of SMTs, minimizing vomiting reflexes is crucial. Dyspeptic patients have increased vomiting sensitivity during EGD [18]. In our hospital, all procedures are performed with sedation. Especially for patients with strong reflexes, nasal endoscopy may be considered [19]. Sedatives alone are known to relieve patient distress, but adding pharyngeal anesthesia to sedatives may decrease the frequency of the vomiting reflex [20]. The use of a modified mouthpiece has also been reported to be effective in controlling the reflex [21, 22].

This study has several limitations: First, it is a single‐center retrospective study, with potential selection bias due to a patient population primarily composed of GERD and achalasia cases, and it lacks a control group for comparison with AEs cases. Second, detailed procedural data of cases without complications, such as procedure time, EPSIS parameters, and final diagnoses, were not assessed in this study. However, since the primary outcome of this study was to elucidate the complication rate of EPSIS, the impact is considered to be minor. Third, owing to the extremely small number of SMT cases, it was not feasible to investigate potential risk factors. Further studies with larger cohorts are needed to validate the safety of EPSIS.

## 5 Conclusions

EPSIS is a safe diagnostic procedure with a complication rate comparable to that of standard EGD. To ensure safety, we recommend performing EPSIS under sedation, using CO₂ insufflation, directly observing GEJ, and controlling insufflation so that the IGP is less than 25 mmHg. However, this threshold may vary depending on the patient's underlying conditions or clinical background; therefore, further research is warranted.

## Ethics Statement

This study was approved by the Showa University Hospital Research Ethics Committee (institutional review board registration no. C‐T2025‐0573).

## Consent

Informed consent was obtained from all patients prior to undergoing EPSIS.

## Conflicts of Interest

Haruhiro Inoue is an advisor for Olympus Corporation and Top Corporation. He has also received educational grants from Olympus Corporation and Takeda Pharmaceutical Co. The other authors have no conflicts of interest to disclose.

## Supporting information



Sup Fig 1.TIF

Sup Fig 2.TIF

Sup Fig 3.TIF
