# Supplementary figures and images for: Complications of Endoscopic Pressure Study Integrated System: Review of 1205 Cases in 10 Years’ Experience
Source: DEN Open. 2025 Jul 16;6(1):e70173. doi: 10.1002/deo2.70173 (PMC12265033; doi:10.1002/deo2.70173)

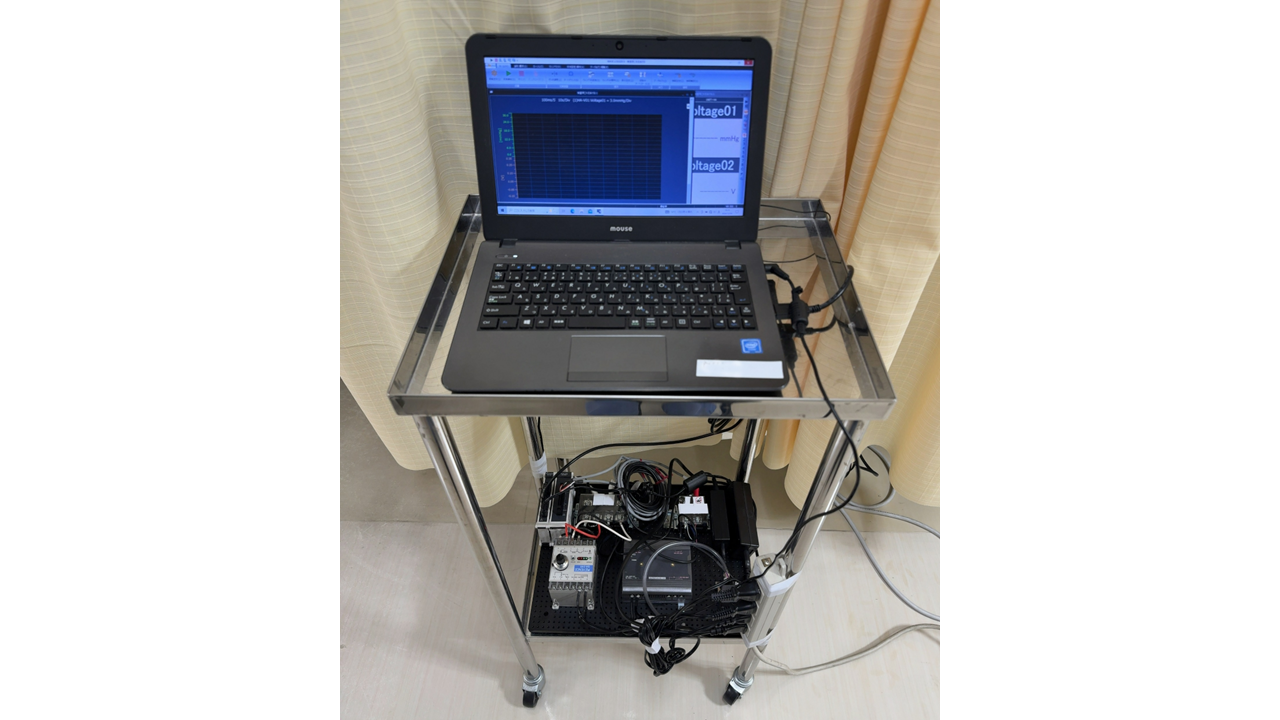

Supplement: Supplementary file 1 — Sup Fig 1.TIF [file DEO2-6-e70173-s002.TIF]

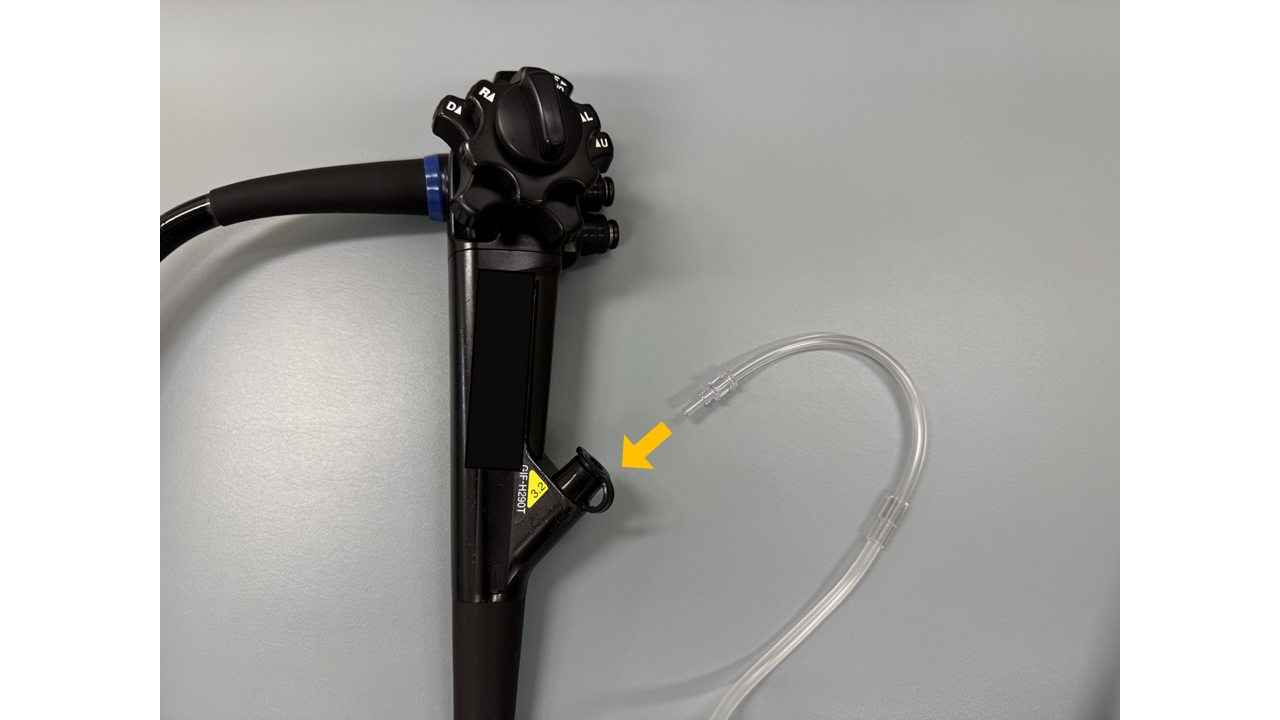

Supplement: Supplementary file 2 — Sup Fig 2.TIF [file DEO2-6-e70173-s003.TIF]

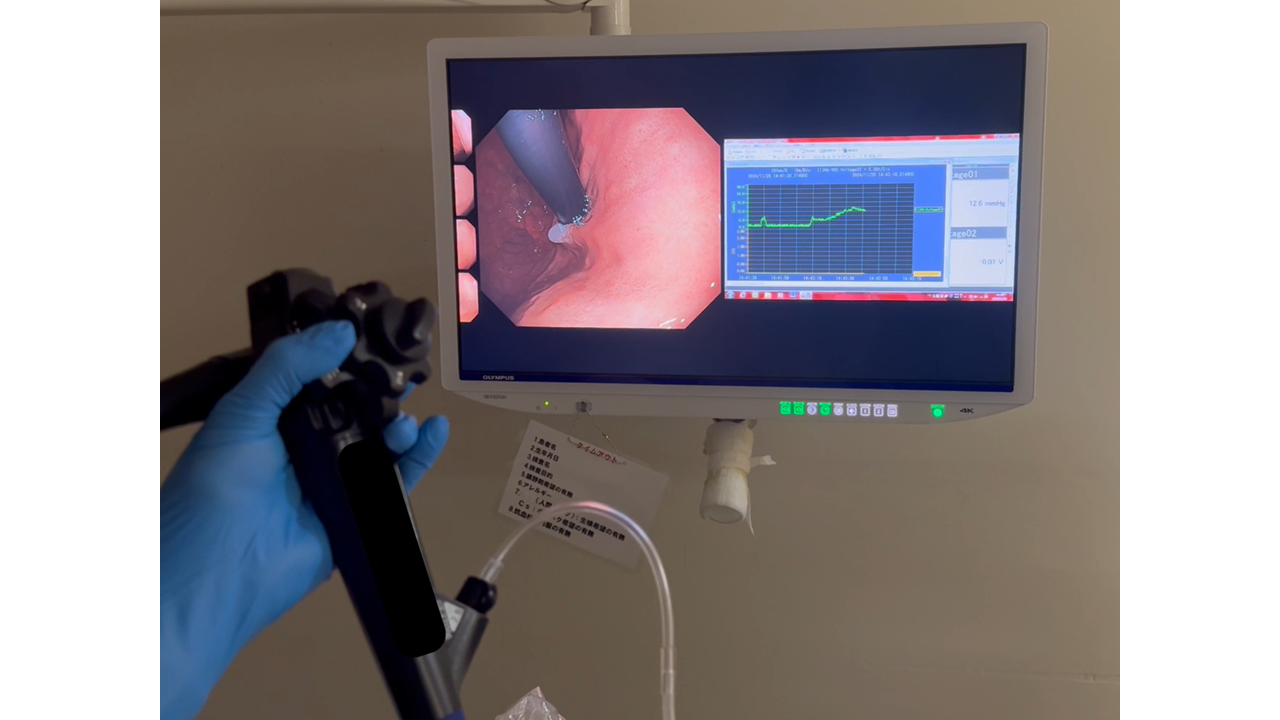

Supplement: Supplementary file 3 — Sup Fig 3.TIF [file DEO2-6-e70173-s001.TIF]
